# Natural occurrences and characterization of *Elizabethkingia miricola* infection in cultured bullfrogs (*Rana catesbeiana*)

**DOI:** 10.3389/fcimb.2023.1094050

**Published:** 2023-03-14

**Authors:** Dongdong Wei, Yuan Cheng, Shuangyan Xiao, Wenyu Liao, Qing Yu, Shuyu Han, Shuaishuai Huang, Jingu Shi, Zongsheng Xie, Pengfei Li

**Affiliations:** ^1^ Guangxi Key Laboratory of Aquatic Biotechnology and Modern Ecological Aquaculture, Guangxi Engineering Research Center for Fishery Major Diseases Control and Efficient Healthy Breeding Industrial Technology (GERCFT), Guangxi Academy of Sciences, Nanning, China; ^2^ China-ASEAN Modern Fishery Industry Technology Transfer Demonstration Center, Beibu Gulf Marine Industrial Research Institute, Guangxi Academy of Marine Sciences, Nanning, China; ^3^ Guangxi Fisheries Technology Extension Station, Nanning, China; ^4^ Guangxi Academy of Fishery Science, Nanning, China

**Keywords:** bullfrog (*Rana catesbeiana*), meningitis-like disease, *Elizabethkingia miricola*, isolation and identification, antimicrobial resistance

## Abstract

**Introduction:**

The bacterium *Elizabethkingia miricola* is a multispecies pathogen associated with meningitis-like disease that has been isolated from several amphibian species, including the bullfrog, but this is the first isolation in Guangxi. In the present study, the dominant bacteria were isolated from the brains of five bullfrogs with meningitis-like disease on a South China farm in Guangxi.

**Methods:**

The NFEM01 isolate was identified by Gram staining; morphological observations; *16S rRNA, rpoB*, and *mutT*-based phylogenetic tree analysis; and physiochemical characterization and was subjected to drug sensitivity and artificial infection testing.

**Results and discussion:**

As a result of identification, the NFEM01 strain was found to be *E. miricola*. An artificial infection experiment revealed that NFEM01 infected bullfrogs and could cause symptoms of typical meningitis-like disease. As a result of the bacterial drug sensitivity test, NFEM01 is highly sensitive to mequindox, rifampicin, enrofloxacin, nitrofural, and oxytetracycline and there was strong resistance to gentamicin, florfenicol, neomycin, penicillin, amoxicillin, doxycycline, and sulfamonomethoxine. This study provides a reference to further study the pathogenesis mechanism of *E. miricola*-induced bullfrog meningitislike disease and its prevention and treatment.

## Highlights

• *E. miricola* was first isolated from bullfrogs in Guangxi.
• *E. miricola* induced meningitis-like disease in bullfrogs.
• *E. miricola* was resistant to the majority of antibiotics tested.

## Introduction

Aquaculture provides humans with over 1/3 of the high-quality protein consumed (Li et al., 2022). The bullfrog is an important aquatic economy native to eastern North America and has been widely introduced worldwide (Akmentins and Cardozo, 2010). With the progress of socio-economic development and increases in people’s standard of living, the demands for food safety and quality keep increasing. The bullfrog is increasingly consumer friendly as a good quality meat, and recently, demand for bullfrog has been increasing (Schloegel et al., 2010; Zhu et al., 2021). The aquaculture of American bullfrogs for the meat industry has expanded worldwide. China, Taiwan, Brazil, and Ecuador are well-known for their significant production, while the United States, France, Canada, Belgium, Italy, and Spain are well-known for their significant consumption (FAO, 2023). The bullfrog is an economical frog, and the development of artificial aquaculture is rapidly developing to meet the rise in market demand (Zhang et al., 2015). The bullfrog was first introduced to China as a food source from Cuba and Japan (Wu et al., 2004). Since then, the cultivation of the bullfrog has made remarkable developments in China and has been introduced to many provinces (Zhang et al., 2015). Recently, there have been frequent occurrences of bullfrog diseases, especially bacterial pandemics, such as epidemic meningitis-like disease (EMD) and red leg syndrome (RLS), which severely damage the bullfrog aquaculture industry (Pasteris et al., 2006; Trimpert et al., 2021). EMD has been frequently occurring in recent years (Zajmi et al., 2022). Disease in the bullfrog is observed as signs of torticollis, head slanting to one side, swimming in circles, and loss of appetite (Hu et al., 2017), and 60-90% of diseased animals die within several days to weeks after the onset (Hu et al., 2020). It is found that the epidemic of bacterial diseases is the main cause of major loss to economic bullfrog farming (Mauel et al., 2002; Li et al., 2018), so in order for frog aquaculture to healthily and rapidly develop, we should accelerate the study of bacterial diseases in aquaculture and find reasonable measures (Yu et al., 2021).

*Elizabethkingia* spp. is a pathogen that threatens the lives of humans and animals (Zajmi et al., 2022). *Elizabethkingia* spp. is a potential zoonotic pathogen (Vancanneyt et al., 1994)*;* it is widely distributed in the natural environment and also exists in the hospital environment (Moore et al., 2016; Chew et al., 2018; Hem et al., 2022). It is a potentially infectious pathogen in the hospital, which can cause newborn meningitis, adult sepsis, and skin and soft tissue infection, and mortality is rather high in infected patients (Furyk et al., 2011). *Elizabethkingia* spp. infection has been reported worldwide, especially in patients whose immune function is compromised, causing a fatal human infection (Dziuban et al., 2018).

Besides human infection, *Elizabethkingia* spp. also infects birds (Vancanneyt et al., 1994), dogs (Bordelo et al., 2016), aquaculture animals such as tilapia (Jacobs and Chenia, 2011), catfish (Laith et al., 2017), and many amphibians, including tiger frogs (Xie et al., 2009), spiny frogs (Lei et al., 2019), and northern leopard frogs (Trimpert et al., 2021). According to earlier research, the *Enterobacteriaceae* (including *Proteus vulgaris* and *Proteus mirabilis*), *Pseudomonas aeruginosa, Aeromonas*, and a number of *Staphylococcus epidermidis* strains were considered to be the pathogens causing EMD (Cunningham et al., 1996). Another study identified *E. miricola* as a pathogen of EMD in black-spotted frogs (Hu et al., 2017). A recent study has shown that *E. miricola* was the pathogen isolated from diseased American bullfrogs (*Lithobates catesbeianus*) in farms in the Guangdong province (Liu et al., 2022). There are many reports on EMD, which is the most serious disease for many kinds of cultured frogs in recent years (Mauel et al., 2002; Xie et al., 2009; Lei et al., 2019), but there is no consensus on the pathogen. A widespread outbreak of disease occurred in bullfrogs on different farms in Guangxi, resulting in high mortality and severe economic losses. Nevertheless, the underlying cause of the explosion of bullfrog disease is not clear. In the present study, we investigated the pathogen of the bullfrog, characterized the pathogen, and isolated the main bacterial pathogen in meningitis. The results of the study provide a theoretical reference for further studies on bullfrog dermatology and for helping to prevent and treat EMD during bullfrog farming.

## Materials and methods

### Bacterial isolation

From early May to July 2022, the death rate of cultured bullfrogs was high in Nanning, Guangxi, China. The bullfrogs were raised within a simple fence of 20 square meters. During this time, the water temperature was between 30 and 33 °C. The bullfrogs were fed twice a day with commercial feed (Tongwei Biotechnology Co., Nanning, China). The water in the housing was removed and replaced with fresh water and a continuous flow of fresh water was provided every day.

The outbreak of disease on the farm caused a high number of deaths among bullfrogs. Five bullfrogs with typical symptoms (weight 107.6 ± 3.2 g per bullfrog) and those who were close to death were chosen for the isolation of pathogenic bacteria according to the previous method (Lei et al., 2019). The heads of the bullfrogs were dissected, and the brain tissue was removed. The brain tissue was then put into a sterile homogenizer, and the appropriate amount of sterile water was added and thoroughly homogenized. The 100-μL homogenate was diluted 10 times and placed on an LB plate and Columbia blood agar plates (Huankai Microbial, Guangzhou, China), which were incubated aerobically and anaerobically at 37 °C overnight. The colonies were selected according to their morphological characteristics and labeled on the LB plate. The strains were expanded and identified. The purified bacterial strain was used for Gram staining, morphological observation, physiological and chemical analysis, molecular identification, and subsequent infection.

### Morphological observation

Several bacteria from different frogs were examined and all properties seemed equivalent, so a colony was selected randomly, and the purified NFEM01 strain was cultured for 48 h. After gram staining, the NFEM01 strain was observed under an optical microscope. Hemolytic activity was determined on a Columbia blood agar plate. The NFEM01 strain was dehydrated by ethanol (25%, 50%, 75%, 100%) for 30 minutes then dried, gold-plated, and visualized using a Hitachi s-3400N (Hitachi, Tokyo, Japan) scanning electron microscope (Bozzola, 2014).

### Physiological and chemical characteristics

Physiological and biochemical characteristics of the NFEM01 strain were analyzed using an API^®^ 20E (bioMérieux, Marcy l’Etoile, France) bacterial identification system. The physiological and biochemical characteristics of the NFEM01 isolate were assessed based on previously published methods (Shayegani et al., 1978).

### *16S rRNA*, *rpoB*, and *mutT* gene sequences and phylogenetic tree analysis

The genomic DNA of the NFEM01 strain was extracted using a bacterial genomic DNA kit (Qiagen, Hilden, Germany). Primers 27 F/1492 R (Weisburg et al., 1991), Eliz rpoB F/Eliz rpoB R, and mutT F/mutT R (**Table 1**) were used to amplify the *16S rRNA* gene, *rpoB* gene (Kenna et al., 2018), and *mutT* gene (Zhang et al., 2020) respectively. Amplified products were detected using 1.5% agarose gel electrophoresis. The positive amplification products were sequenced by Aoke Dingsheng Biotechnology Co., Ltd. (Wuhan, China). Gene sequence analysis using the Basic Local Alignment Search Tool (BLAST) (https://blast.ncbi.nlm.nih.gov/Blast.cgi) and a nucleotide sequence identity of > 98% was used as the criterion for identification. All gene sequences are stored in the NCBI GenBank database with the registration number PRJNA893762.

**Table 1:** Sequence of the oligonucleotide primers used for PCR amplifications in this study.

Target Gene	Oligo	Sequence 5′–3′	*Product length (bp)*	Reference
16s RNA	27F	AGAGTTTGATCATGGCTCAG	1465	Weisburg et al., 1991
	1492R	TACGGTTACCTTGTTACGACTT		
rpoB	Eliz rpoB F	CTCCGGAAGGACCAAACATTG	1392	Kenna et al., 2018
	Eliz rpoB R	CAACCGTCCAGTCAGATCC		
mutT	mutT F	CGTATATATGTAGGTCGGAACAG	140	Zhang et al., 2020
	mutT R:	CCATAGAACACAA AACATCAGCA		

### Antibiotic susceptibility test

Antimicrobial susceptibility was tested using the Kirby Bauer disk diffusion method. Bacterial suspensions were uniformly distributed on a Mueller Hinton agar plate (Hangzhou Tianhe Microbial Reagent Co., Ltd., Hangzhou, China). Antibiotic disks were placed on the surface of the culture plate. The plate was incubated at 28°C for 24 h. The inhibition zone was measured, and the results were interpreted according to the Clinical Laboratory Standards Institute (CLSI) standard (2016) and previous research (Jorgensen and Turnidge, 2015).

### Artificial infection test

After 7 days of domestication, 60 healthy bullfrogs (7.07 ± 0.82 g) were divided into six groups. The NFEM01 isolate was cultured in LB liquid at 37°C for 24 h. The bacterial concentration (colony-forming units) CFU·mL^-1^ was determined by LB plate counts after the strain had been subjected to 10-fold serial dilution. A group of 10 bullfrogs was infected by a 0.2 ml intraperitoneal injection with 5.18 × 10^4^, 5.18 × 10^5^, 5.18 × 10^6^, 5.18 × 10^7^, 5.18 × 10^8^, 5.18 × 10^9^ CFU·mL^-1^ of NFEM01, respectively. Phosphate buffered saline (PBS) was the negative control. Clinical symptoms and mortality from infection to 14 days post-infection were recorded. The brains of the dead bullfrogs were collected to reisolate NFEM01.

### Histopathological observation

The samples of the liver, spleen, kidney, intestine, and brain of the bullfrog from the pathogenicity study were fixed in 10% buffered formalin, trimmed, dehydrated using ethanol, and embedded in paraffin blocks for histopathological examination. These blocks were sectioned and stained with hematoxylin and eosin (H&E).

### Statistical analysis

Data were analyzed in the statistical program GraphPad Prism version 5 (GraphPad Software, San Diego, California, USA) using one-way analysis of variance (ANOVA) followed by Tukey’s multiple comparison test. *P* < 0.05 were considered significant differences.

## Results

### Bullfrogs with meningitis-like disease

**Figure 1:**
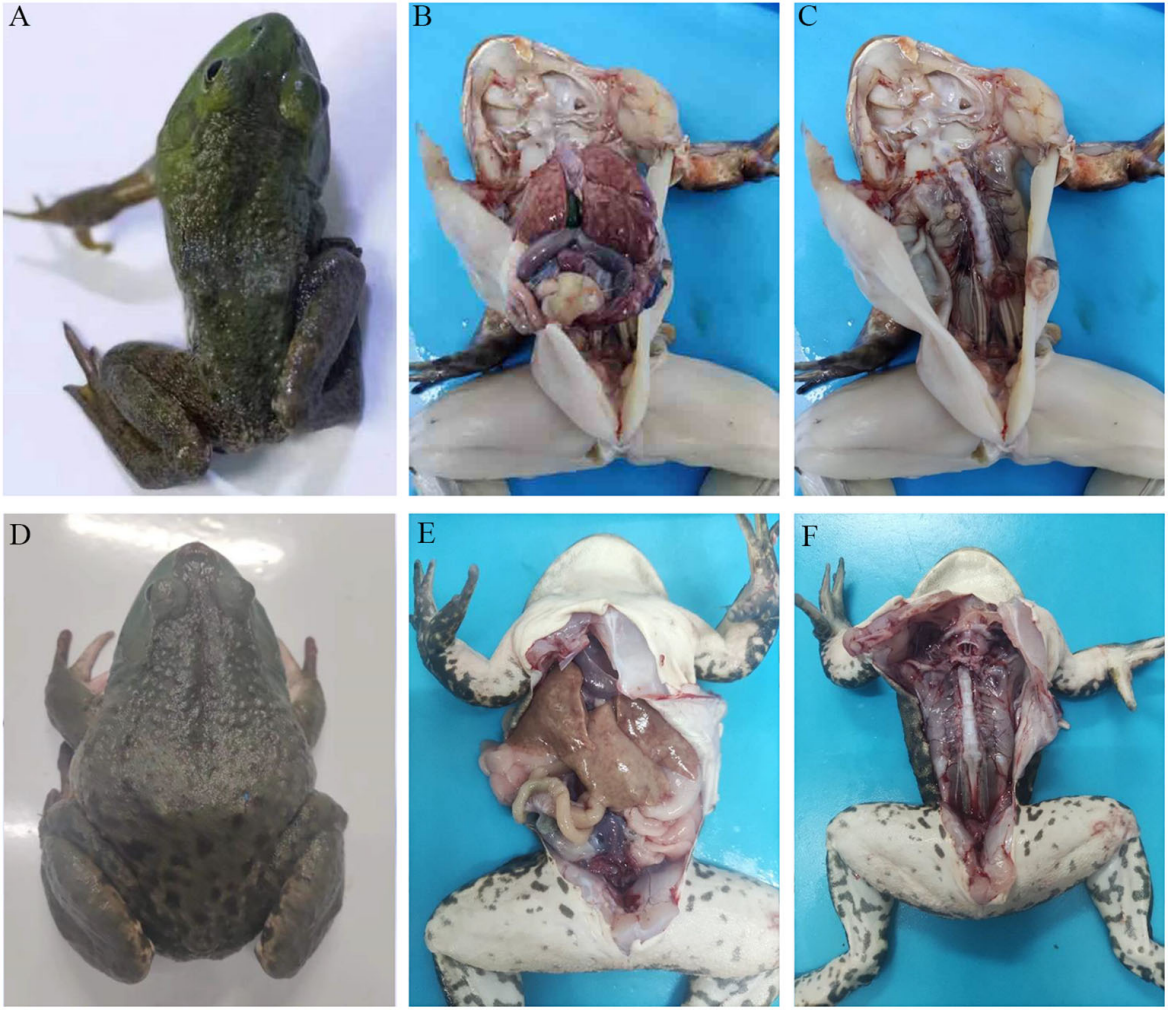
Clinical features of a bullfrog epidemic meningitis-like disease. The diseased bullfrogs showed serious torticollis **(A)**, the liver, spleen, and kidney were enlarged **(B)**, and the spine of one frog was curved **(C)**. The healthy bullfrog neck **(D)**, liver, spleen, kidney **(E)**, and spine **(F)** are shown.

The diseased bullfrogs showed signs of severe neurological disorder (**Figure 1A**), the liver, spleen, and kidney were enlarged (**Figure 1B**), and the spine of a frog was curved (**Figure 1C**). The healthy bullfrog neck (**Figure 1D**), liver, spleen, kidney (**Figure 1E**), and spine (**Figure 1F**) are also shown.

### Morphological observation

**Figure 2:**
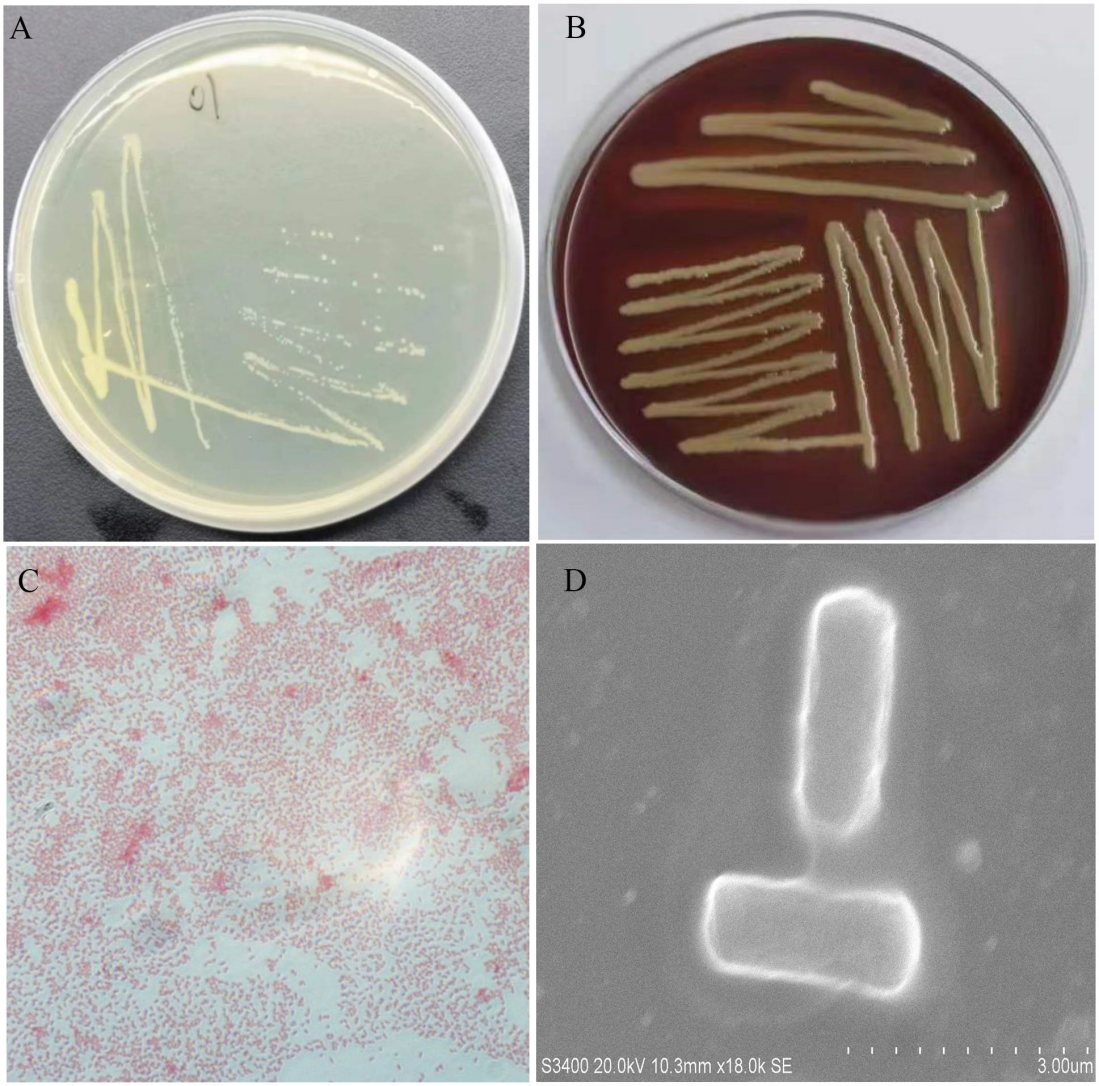
Morphological features of NFEM01 isolated from the occurrence of bullfrog disease cultured in Guangxi. **(A)** NFEM01 single colonies, **(B)** the hemolytic activity of the strain was determined according to the hemolytic area around the colony, **(C)** Gram stained under a light microscope, and **(D)** individual cells under a scanning electron microscope.

All five diseased bullfrogs were shown to contain one bacterial type for which there was heavy growth on LB plates, with very little growth of other organisms. The predominant bacterial type found on aerobic plates and the colonies were selected. After incubation at 37°C for 24 h, the colonies appeared smooth, raised, round, and white (**Figure 2A**). The NFEM01 strain was a gram-negative bacterium (**Figure 2C**). Appearance after growth on Colombia blood agar (**Figure 2B**) and following scanning electron microscopy (**Figure 2D**) are shown. The NFEM01 had a clear transparent zone around the colonies on the blood agar plates indicative of beta hemolysis activity. Scanning electron microscopy showed that the bacteria were nearly rod-shaped and approximately 1.4 µm in diameter and 2.6 µm in length.

### Physiological and chemical characteristics

The results of the physiological and chemical characteristics of NFEM01 are shown in **Table 2**. The NFEM01 isolate was negative for glucose, lactose, maltose, and mannose utilization but was positive for honey disaccharide, cellulose disaccharide, xylose, arabinose, rhamnose, and sucrose. The hydrolysis of urea was positive, while that of citrate was negative.

**Table 2:** Physiological and biochemical characteristics of *NFEM01*.

Item	*NFEM01*	*E. miricola* (Huang et al, 2019)	*E. miricola* (Lei et al, 2019)
ONPG	+	ND	+
Arginine decartobxylase	+	–	ND
Lysine decarboxylase	–	–	ND
Omithin decarboxylase	–	–	ND
Citrate-sodium	–	ND	ND
H_2_S production	–	–	–
Urease	+	–	+
Tryptophan deaminase	–	–	ND
Indole production	+	N	+
Voges-Prokaver	–	–	ND
Gelatinase	+	ND	+
Glucose	+	ND	+
Mannitol	–	ND	+
Inositol	–	ND	ND
Sorbitol	–	ND	+
Rhamnose	–	ND	–
Sucrose	–	ND	+
Melibiose	–	ND	–
Amygdalin	–	ND	ND
Arabinose	–	N	–
Oxidase	+	ND	+
NO_2_	–	ND	ND
N_2_	–	ND	ND
MOB	+	–	–
McC	ND	ND	ND
OF-O	ND	ND	ND
OF-F	ND	ND	ND

+, positive reaction; -, negative reaction; N, not applicable; V, variable reaction; (+), weak or delayed reaction; ND, not determined.

### Molecular identification

**Figure 3:**
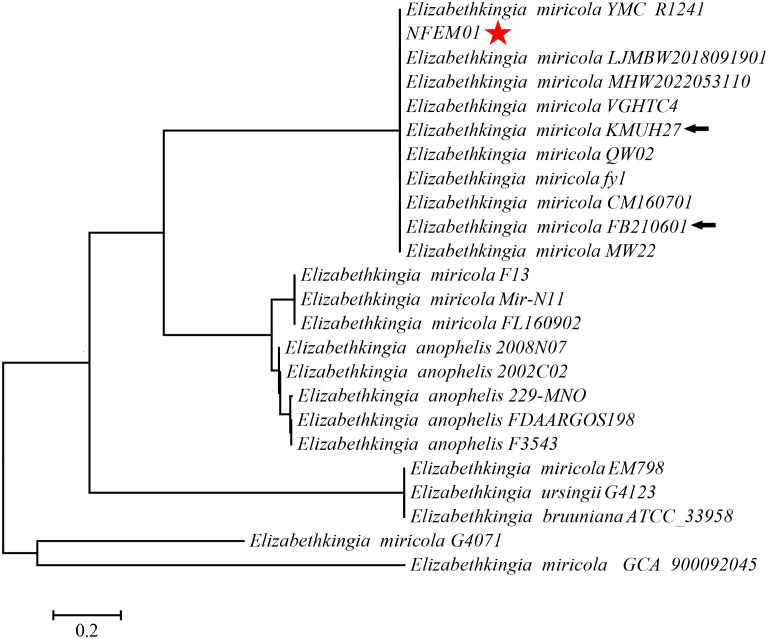
A phylogenetic tree was constructed based on the *16S rRNA* gene of NFEM01 by neighbor-joining method and displays the percentage of bootstrap values on each branch point (1000 copies). The scale represents 0.02 nucleotide substitutions each site. Star refers to the strain from this study. The arrow stands for the type strain of the species.

**Figure 4:**
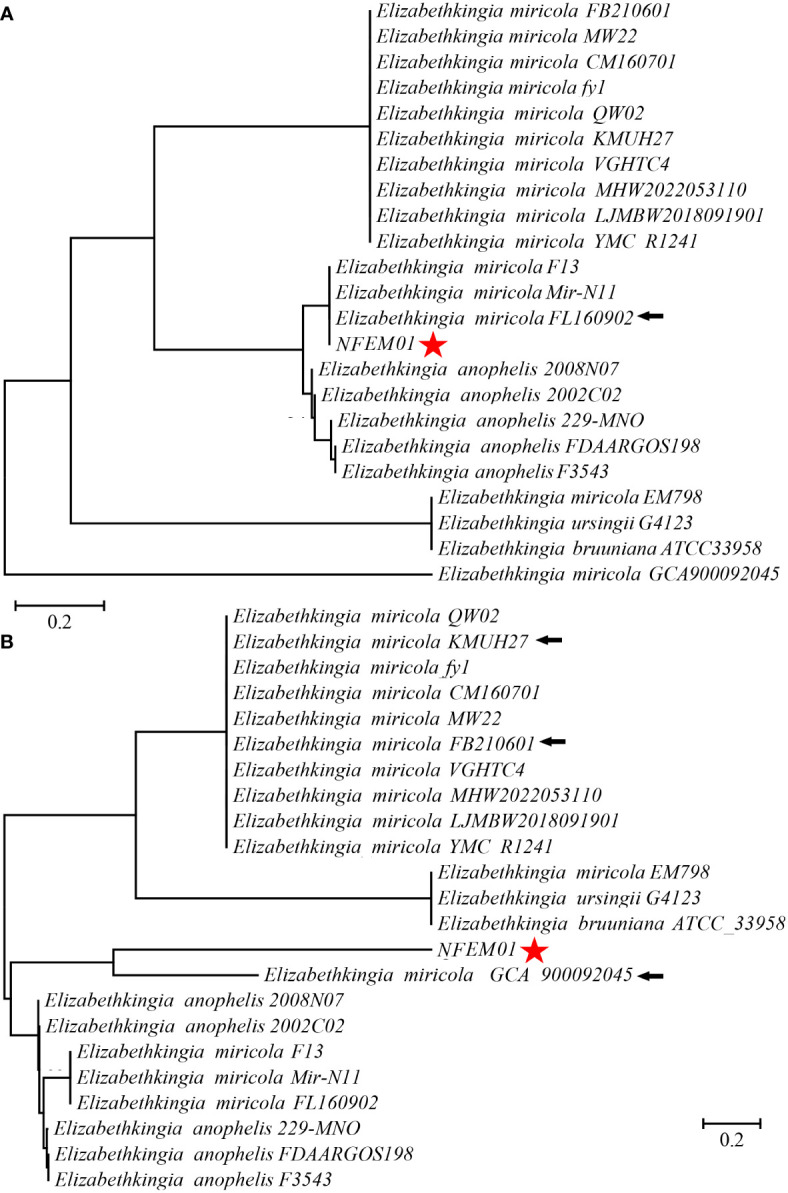
**(A)** Phylogenetic tree analysis of NFEM01 using the *rpoB* gene. **(B)** Phylogenetic tree analysis of NFEM01 using the *mutT* gene. Star refers to the strain from this study. The arrow stands for the type strain of the species.

Portions *16S rRNA*, *rpoB*, and *mutT* gene sequences were amplified from the isolate, sequenced, and the latter submitted to GenBank with the registration number PRJNA893762. Analysis of *16S r RNA*, *rpoB*, and *mutT* genes sequences by BLAST in NCBI was performed and sequences with high sequence identity were identified. According to phylogenetic characteristics, together with *16S rRNA* (**Figure 3**), *rpoB* (**Figure 4A**), and *mutT* (**Figure 4B**) gene sequence analysis, the sequences of these genes were clustered with *E. miricola* and showed 98.86%, 99.85%, and 100% similarity to the FB210601, FL160902, and FL160902 strains, respectively. The NFEM01 was identified as *E. miricola*.

### Antibiotic susceptibility

The results of the drug sensitivity test for 12 antibiotics showed that the isolated NFEM01 was highly sensitive to mequindox, rifampicin, enrofloxacin, nitrofural, and oxytetracycline but showed strong resistance to gentamicin, florfenicol, neomycin, penicillin, amoxicillin, doxycycline, and sulfamonomethoxine (**Table 3**).

**Table 3:** Drug sensitivity test results of NFEM01 strain.

Drugs names	Inhibition zone diameter (mm)	Sensitivity
Gentamicin	6	R
Florfenicol	9	R
Enrofloxacin	31	S
Nitrofural	26	S
Oxytetracycline	32	S
Neomycin	15	R
Mequindox	40	S
Penicillin	6	R
Amoxicillin	8	R
Rifampicin	24	S
Doxycycline	6	R
Sulfamonomethoxine	6	R

Susceptible (S), Intermediate (I), Resistant (R).

### Artificial infection

**Figure 5:**
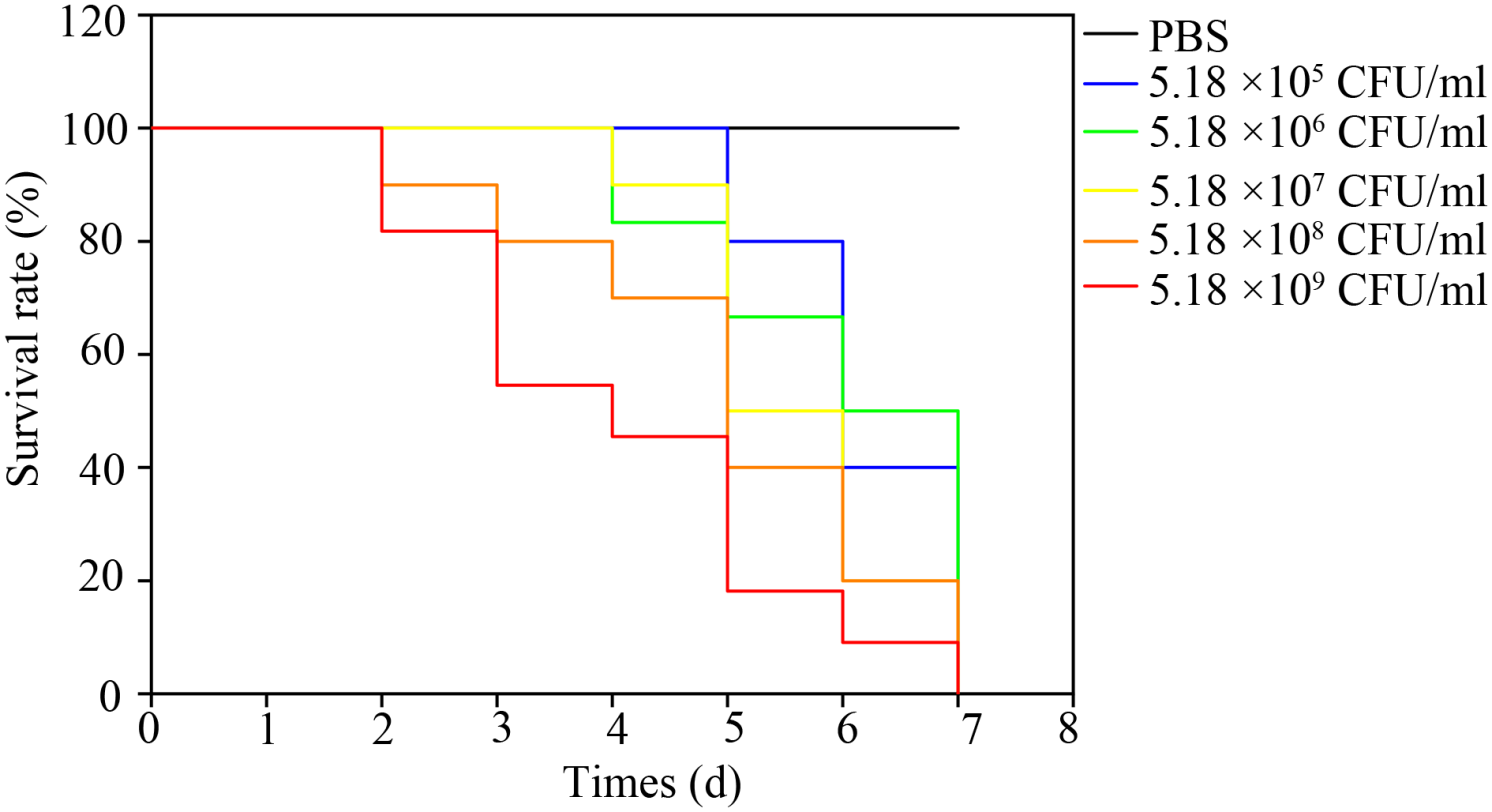
Kaplan-Meier survival curves of bullfrogs infected with different doses of NFEM01. Control group/phosphate-buffered-saline (PBS), the concentrations in the infection group were 5.18 × 10^4^, 5.18 × 10^5^, 5.18 × 10^6^, 5.18 × 10^7^, 5.18 × 10^8^, 5.18 × 10^9^ CFU·mL^-1^ NFEM01.

The bullfrogs began to die 2 days after the highest dose of artificial infection (5.18 × 10^9^ CFU·mL^-1^). High mortality occurred 3 days after inoculation of 5.18 × 10^5^, 5.18 × 10^6^, 5.18 × 10^7^, 5.18 × 10^8^, 5.18 × 10^9^ bacteria. The bullfrog mortality rates were 60%, 60%, 100%, 100%, and 100%, respectively (**Figure 5**). The survival rate of the highest dose group was significantly lower than that of the control group (*P* < 0.05). Mortality was not observed in the control group. Bullfrog death after artificial infection is similar to natural pathogen-induced death, including congestion and hemorrhage of the skin of the abdomen and hind limb, spleen swelling, ascites, liver swelling, and gastrointestinal congestion. NFEM01 was isolated from all dead infected bullfrogs, while NFEM01 was not isolated from the control bullfrogs.

### Histopathological observation

**Figure 6:**
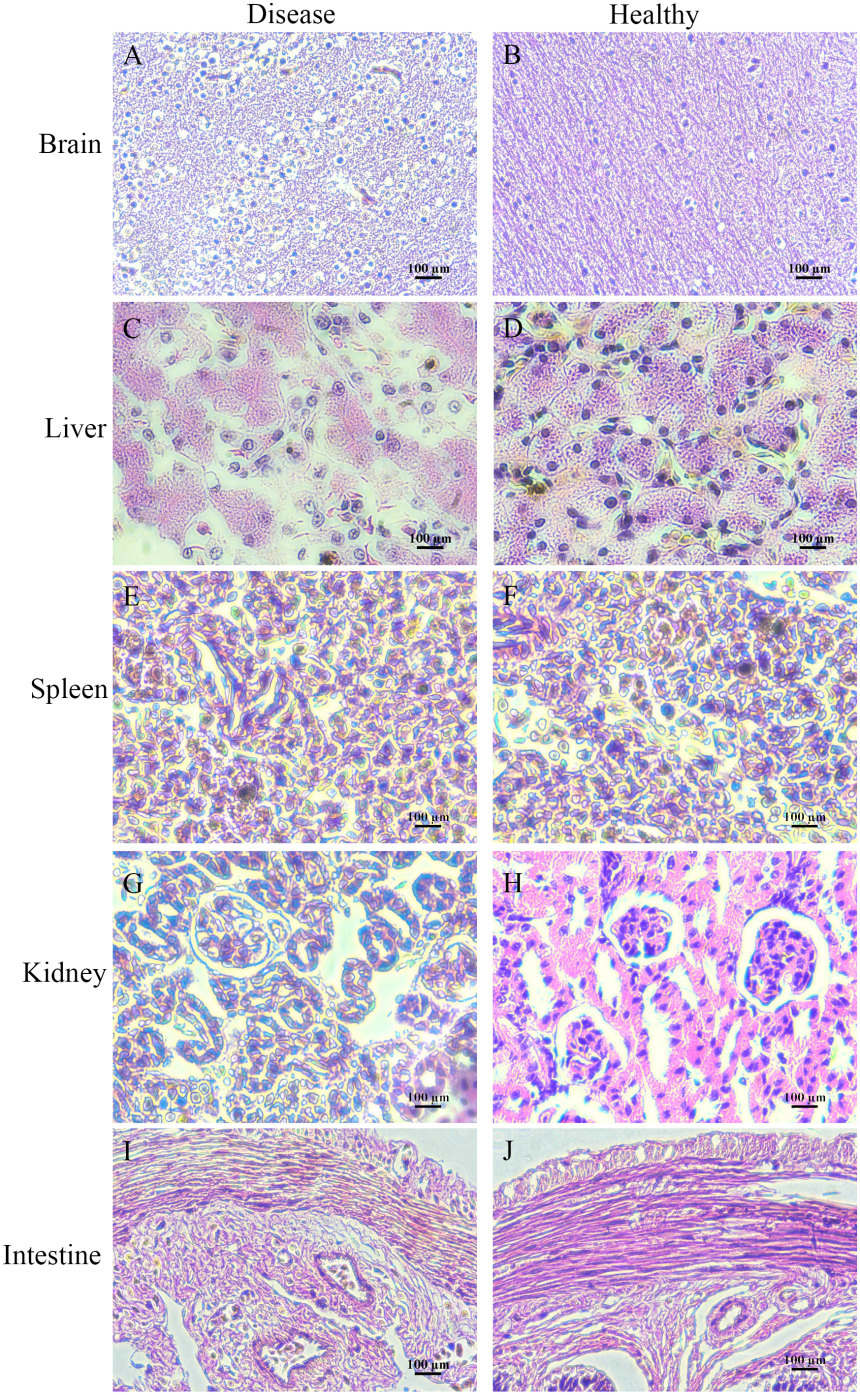
Histologically diseased bullfrog liver **(C)**, spleen **(E)**, kidney **(G)**, intestine **(I)**, and brain **(A)** were observed in comparison with tissue sections of healthy bullfrog liver **(D)**, spleen **(F)**, kidney **(H)**, intestine **(J)**, and brain **(B)**, where the most obvious signs of brain injury are ventricular thickening, membranous tissue degeneration, and a sharp increase in glia, showing the pathological changes typical of encephalitis and meningitis.

Histologically, the pathological changes of diseased bullfrogs in the brain (**Figure 6A**), liver (**Figure 6C**), spleen (**Figure 6E**), kidney (**Figure 6G**), and intestine (**Figure 6I**) were observed, and the most obvious brain lesions were the thickening of the ventricles, the degeneration of the membrane tissue, and the sharp increase of neuroglia, showing typical pathological changes of encephalitis and meningitis, compared with the healthy bullfrog brain (**Figure 6B**), liver (**Figure 6D**), spleen (**Figure 6F**), kidney (**Figure 6H**), and intestine (**Figure 6J**).

## Discussion

Many studies have shown that *E. miricola* can cause clinical meningitis in addition to pneumonia and meningitis in newborns, children, and the elderly (Dziuban et al., 2018). The immunocompromised are particularly at high risk. In addition to humans and poultry, *E. miricola* bacteria also infect many aquatic animals (Jacobs and Chenia, 2011; Laith et al., 2017). In recent years, *E. miricola* bacteria have become an emerging pathogen of frog farming, especially bullfrog farming (Liu et al., 2022). The infected bullfrog shows disease symptoms such as severe torticollis; curvature of the spine; and enlargement of the liver, spleen, and kidneys, which are also reported in several other frog species (Xie et al., 2009; Lei et al., 2019). This phenomenon also occurs in black-spotted frogs (Hu et al., 2017). In the current study, the NFEM01 strain was isolated from the brain of bullfrogs with EMD in Nanning, Guangxi. The surface of a typical NFEM01 colony was smooth and wet and the edge was neat and white, which was consistent with *E. miricola* isolated from the spiny frog (Lei et al., 2019). NFEM01 has beta-hemolytic activity, which indicates that NFEM01 has strong pathogenic potential.

In the API^®^ 20E test, the NFEM01 strain showed similar characteristics to those reported previously (Huang et al., 2019), but differences between isolates of *Elizabethkingia* spp. in trypsin response were observed. Some of the characteristics of *Elizabethkingia* spp. have been summarized. They can all produce catalase, phosphatases, galactosidases, and indole, whereas they cannot hydrolyze starch, use malonate, and ferment galactose, sorbitol, inositol, and salicylic acid. However, some features are variable in the same species and the phenotypic similarities between known species challenge the correct identification of clinical isolates (Nicholson et al., 2018). The genus *Elizabethkingia* is genetically heterogeneous, and the identification by phenotypic similarity is challenging for the accurate identification of clinical isolates (Bruun and Ursing, 1987).

In previous works, the *16S rRNA* gene was used for the clinical reports of most cases (Frank et al., 2013; Chew et al., 2018). However, there are five copies of the *16S rRNA* gene in *Elizabethkingia* spp., and there are some differences between them (Nicholson et al., 2018). Therefore, it is difficult to distinguish *Elizabethkingia* spp. from the *16S rRNA* gene sequence alone, so other methods to support identification are needed (Chew et al., 2018; Lei et al., 2019). The *rpoB* gene sequencing is superior to other gene targets because it has a higher resolution than *16S rRNA* gene sequencing, and is used for delineating new bacterial species (Adékambi et al., 2009; Turton et al., 2010). Nicholson et al. (2018) first proposed using the *rpoB* gene to identify *Elizabethkingia* species. Subsequently, Kenna et al. studied the distribution of *Elizabethkingia* species using *rpoB* gene sequencing. In their study, 43 isolates from 38 patients formed a cluster with *E. miricola* and *E. bruuniana* sp. *nov*. (Kenna et al., 2018). In a separate study based on *16S rRNA* and *rpoB* gene sequencing, the authors identified six patients infected with *E. bruuniana* between 2005 and 2017 (Lin et al., 2019). On this basis, we further verified and confirmed the *mutT* gene of *E. miricola* by amplification and sequencing. Zhang et al. (2015) established a real-time fluorescent quantitative PCR system based on *mutT* gene amplification, which could specifically identify *E. miricola* and had no nonspecific amplification with many bacteria. In our study, a combination of *16S rRNA, rpoB*, and *mutT* genes was used to identify *E. miricola* isolated from the bullfrog, eliminating a possible error caused by single *16S rRNA*-based identification.

Previous antibiotic susceptibility testing revealed *E. miricola* was resistant to erythromycin and oxytetracycline (Colapietro et al., 2016; Han et al., 2017; Gao et al., 2021). *Elizabethkingia* spp. is highly resistant to various antibiotics, leading to fewer choices of therapeutic drugs. Because of this, clinically, patients with *Elizabethkingia* spp. infections have high mortality (Opota et al., 2017). In this study, the NFEM01 was resistant to the majority of antibiotics, including gentamicin, florfenicol, neomycin, penicillin, amoxicillin, doxycycline, and sulfamonomethoxine. Therefore, the choice of antibiotics to treat EMD is limited. The NFEM01 isolate showed high resistance to multiple antibiotics similar to previously reported isolates from the Chinese spiny frog (Lei et al., 2019). The main reason for the multi-drug resistance of *Elizabethkingia* spp. is that there are many natural resistance genes on its chromosome, which can produce antibiotic-inactivating enzymes and lead to corresponding antibiotic resistance, for example, Metallo-β-lactamases (MBLs) (Opota et al., 2017). The use of Chinese herbal medicines may be a potentially effective approach (Li et al., 2021).

In our study, the mortality of the infected bullfrog was 40% and 100%, respectively when 10^5^ and 10^7^ CFU·mL^-1^ NFEM01 were injected. These results are similar to those of another study, which found that the mortality rate of infected black-spotted frogs was 50% and 70% when injected with *E. miricola* FL160902 at 10^7^ and 10^8^ CFU·mL^-1^, respectively (Hu et al., 2017). Another study indicated that the mortality rates of Chinese spiny frogs after infection at 10^6^, 10^7^, and 10^8^ CFU·mL^-1^ were 50%, 80%, and 100%, respectively (Lei et al., 2019). These traits suggest that the strains isolated in these studies all show strong pathogenicity to frogs. However, in previous studies, mortality after infection at 10^8^ CFU·mL^-1^ was 80% and 33.3%, with relatively low lethality in the black-spotted frog (Huang et al., 2019). These studies suggest that different bacterial strains from amphibians may have different pathogenicity or that different amphibian species have differing susceptibility.

## Conclusion

In summary, *E. miricola* was confirmed as the pathogenic bacterium isolated from the brain of bullfrogs with meningitis-like disease. *E. miricola* was first isolated from the bullfrog in Guangxi and is highly pathogenic to bullfrogs. This provides a reference for further study of the pathogenesis mechanism, propagation, and prevention of the disease. 

## Data availability statement

The datasets presented in this study can be found in online repositories. The names of the repository/repositories and accession number(s) can be found in the article/supplementary material.

## Ethics statement 

The animal study was reviewed and approved by the ethics committee of Guangxi Academy of Sciences.

## Author contributions

PL, DW, and ZX conceived and designed the research. YC, SX, WL, QY, SYH, SSH, and JS performed the experiments and analyzed the data. DW wrote the manuscript. All authors contributed to the article and approved the submitted version.
